# Signet ring cell variant of follicular thyroid carcinoma: Report of two cases with focus on morphological, expressional and genetic characteristics

**DOI:** 10.1186/s13000-019-0904-3

**Published:** 2019-11-07

**Authors:** Martin Hysek, Kenbugul Jatta, Adam Stenman, Eva Darai-Ramqvist, Jan Zedenius, Anders Höög, C. Christofer Juhlin

**Affiliations:** 1 Department of Oncology-Pathology, BioClinicum J6:20, Karolinska Institutet, 171 64 Stockholm, Sweden; 2 Department of Pathology and Cytology, Karolinska University Hospital, Stockholm, Sweden; 3 Department of Molecular Medicine and Surgery, Karolinska Institutet, Stockholm, Sweden; 4 Department of Breast, Endocrine Tumors and Sarcoma, Karolinska University Hospital, Stockholm, Sweden

**Keywords:** Signet ring cell, Follicular thyroid cancer, PTEN

## Abstract

**Background:**

Follicular thyroid carcinoma (FTC) is a neoplasm that presents with a micro-follicular growth pattern and a neutrally stained cytoplasm. Seldom, FTCs display unusual morphological characteristics – but given the rarity of these histological subtypes, little is known regarding the underlying genetics and the coupling to patient outcome.

**Case presentation:**

We present two extremely rare cases of minimally invasive FTC with signet ring cell morphology (SRC-FTC) and describe the cytological, microscopic, immunohistochemical and molecular features for both tumors. Both were male patients, age 71 and 51 respectively. The preoperative cytology for both cases could not pinpoint a clear-cut signet ring cell morphology, but a tendency towards nuclear marginalization was seen. The tumors were 38 mm and 22 mm respectively and displayed evident signet ring cell features in subsets of tumor cells as well as degenerative stromal changes. The tumor cells were positive for TTF1, PAX8 and thyroglobulin, and the proliferation indexes were 4% and 1,9% respectively. Both tumors displayed capsular invasion, but not lymphovascular invasion. The tumors were sequenced for mutations in the *TERT* promoter and 22 additional cancer-related genes, interestingly; one patient was shown to carry a deleterious intronic variant in *PTEN*, a tumor suppressor gene coupled to thyroid tumorigenesis and Cowden syndrome. Both patients are alive and well awaiting postoperative radioiodine treatment.

**Conclusions:**

The SRC-FTCs described herein were small, *TERT* promoter wildtype tumors exhibiting low proliferation, thereby suggesting that these exceedingly rare lesions probably carry a favorable prognosis – although the scarce availability regarding descriptions of this tumor entity nevertheless might justify careful clinical monitoring and mandate investigations in larger case series.

## Background

Follicular thyroid tumors constitute the most common thyroid neoplasia, and are categorized as follicular adenomas (FTAs), follicular tumors with uncertain malignant potential (FT-UMPs) and follicular carcinomas (FTCs). The FTCs are furthermore split into three different groups based on histological features: minimally invasive FTCs, encapsulated angio-invasive FTCs and widely invasive FTCs [1]. Regularly, follicular thyroid tumors are seen with a neutrally stained cytoplasm, but subsets of tumors present with an eosinophilic cytoplasm (oncocytic cell tumors). More uncommonly encountered histological variants include follicular tumors with clear-cell or signet ring cell (SRC) morphology, two poorly characterized entities based on the scarce literature available [2–8].

The SRC appearance in tumors is most intimately coupled to gastric carcinoma, but the phenotype is also reported in adenocarcinomas from other primary sites [9]. The morphological hallmark is defined as a vacuolization of the cytoplasm that marginalizes the nucleus. The causes underlying the SRC morphology include interactions between mucin 4 (MUC4) and ErbB2/Her2, leading to a constitutively active stimulation of cell growth as well as mucin vacuolization [10, 11].

In the thyroid gland, SRC features derive from either accumulations of mucin, lipids, glycogen and thyroglobulin, and have been described for secretory carcinoma, papillary thyroid cancer (PTC), noninvasive follicular thyroid neoplasm with papillary-like nuclear features (NIFTP), FTA and FTC [3–6, 8]. The description stems from single case reports and small case series with less than 30 reported SRC cases in total - of which FTCs with this phenotype constitute seven cases.

The underlying molecular alterations of these lesions have only been partly dissected. In a previous study of thyroid SRC tumors, single *PTEN* and *FGFR3* mutations were found in an FTA and NIFTP respectively, as well as an *ETV6*-*NTRK3* fusion in a secretory carcinoma of the thyroid [6].

In this study, two exceedingly rare cases of minimally invasive follicular thyroid carcinomas presenting with SRCs are detailed from cytological, histological and molecular aspects.

## Case presentation

### Case 1

**Fig. 1:**
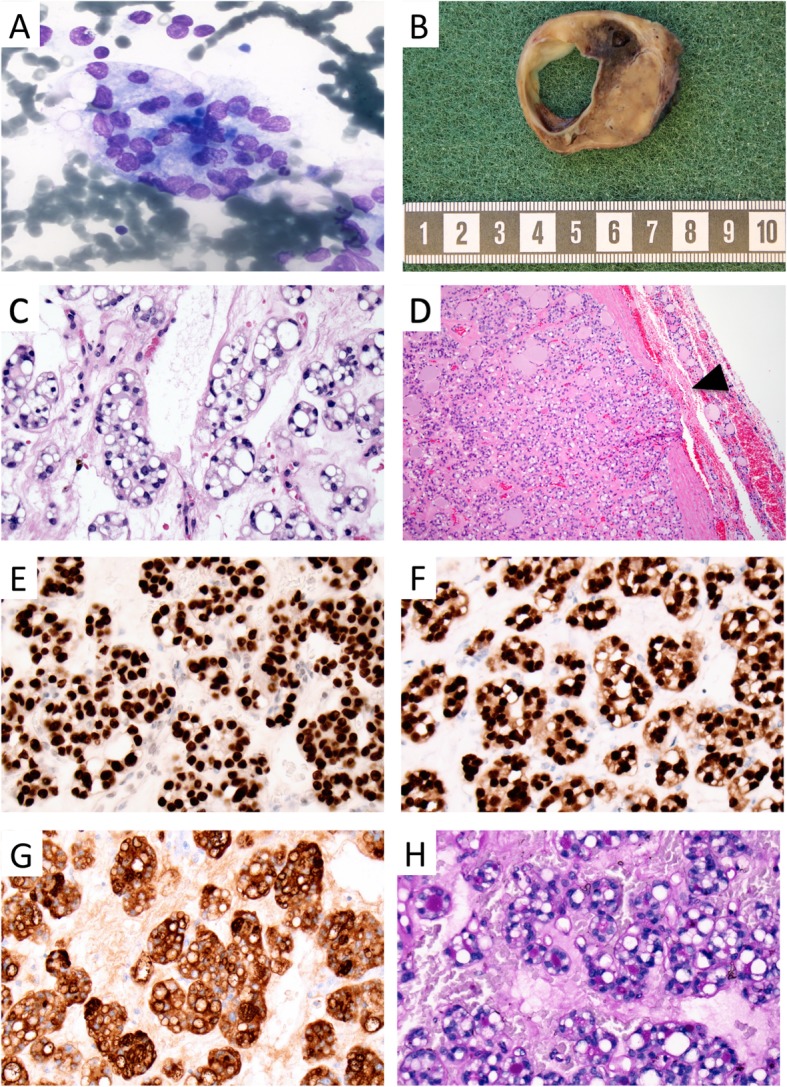
Cytological, macroscopic and histological illustrations of case 1. **a** Cytological preparation from the thyroid lesion at ×1000 magnification depicting thyrocytes with large, irregular nuclei with suggestive nuclear marginalization in subsets of cells. No clear-cut signet ring cell phenotype was evident. **b** Macroscopic view of a coronary section through the thyroid lesion, visualizing a 38 mm encapsulated tumor with a tan cut surface, focal hemorrhages and a central cystic clearing. **c** H&E staining of the thyroid tumor at × 400 magnification, portraying thyrocytes with cytoplasmic vacuolization and nuclear marginalization (signet ring cell appearance) in the majority of tumor cells. **d** Representative focus depicting capsular invasion, marked with an arrowhead, × 100 magnification. **e-g** Immunohistochemical analyses at × 400 magnification; TTF1 nuclear stain (**e**), PAX8 nuclear stain (**f**), thyroglobulin cytoplasmic stain, including subsets of vacuoles (**g**). **h:** Histochemical PAS stain at × 400 magnification, note how the cytoplasmic vacuoles stain negative

The patient is a 71-year old man of Swedish ethnicity without family history of thyroid related diseases. Previous medical conditions include paroxysmal atrial fibrillations, hypertension, a cerebrovascular lesion and malignant melanoma. In 2018, the patient was hospitalized for a minor trauma, and a CT scan of the thorax fortuitously visualized a thyroid lesion, measuring 50 mm. The patient was clinically euthyroid. A cytological fine needle aspiration (FNA) biopsy was performed, and the diagnosis was consistent with a follicular neoplasm, Bethesda IV, with a low proliferation count of 2% as determined by a cytological Ki-67 index. Following a retrospective evaluation, subsets of cells displayed suggestive nuclear marginalization, but no clear-cut signet ring cell appearance was noted (Fig. 1a). The patient was referred to our department, and ultrasonographic examination of the neck revealed a 42 mm, partly cystic nodule in the caudal aspects of the left thyroid lobe. The patient underwent a diagnostic thyroidal lobectomy in September the same year.

The left thyroid lobe measured 70 × 50 × 30 mm and displayed a weight of 50 g. At gross examination, a demarcated and encapsulated lesion measuring 38 × 30 × 36 mm with a tan cut surface, focal hemorrhages and a central cystic clearing of 25 mm was observed (Fig. 1b). Histological examination revealed a circumscribed lesion consisting of sparsely to densely cellular areas against a background of a fibrous and hemorrhagic stroma. The cells were growing in a predominant micro-follicular pattern, and exhibited enlarged, oval to irregularly shaped nuclei with a finely dispersed chromatin and prominent nucleolus. No nuclear features suggestive of papillary thyroid carcinoma were seen. In the majority of cells, the cytoplasm was observed with a balloon-like vacuolized dilation with an accompanied marginalization of the nucleus, corresponding to signet ring cell morphology (Fig. 1c). No mucinous deposits in the surrounding stroma were seen. Tumor necrosis was not present, and the mitotic count was not elevated. Several areas with capsular invasion were noted, but no focus with vascular invasion was observed (Fig. 1d).

The tumor cells were positive for cytokeratin MNF116, cytokeratin 7, TTF1, PAX8 and thyroglobulin (Fig. 1e-g, Table 1). A subset of the vacuoles stained positive for thyroglobulin but were negative for periodic-acid-schiff (PAS) and PAS-diastase stains (Fig. 1h). MUC5Ac was seen with focal and weak positive staining, but in similar intensities as the surrounding normal thyroid tissues. The tumor cells were negative for cytokeratin 20, CDX2, calcitonin, PTH, MUC2, MUC4, MUC6 and mucicarmine. The Ki-67-index was 4%. Differential diagnoses such as medullary thyroid carcinoma, metastatic signet ring cell carcinoma of the GI tract, parathyroid carcinoma, primary mucinous carcinoma and mammary analog secretory carcinoma were ruled out.

**Table 1:** Clinical and histopathological charateristics of the two patients with SRC-FTC

										*Immunohistochemical markers*					
Case no.	Gender	Age at surgery	FNA diagnosis	Histo-pathological diagnosis	SRC component (%)	Vascular or capsular invasion	Tumor size (mm)	ETE	pTNM v8	TTF1	THYR	PAX8	CDX2	PDX1	Ki-67%
1	M	71	Bethesda IV	miFTC	> 50	Capsular	38	No	pT2Nx	+	+	+	–	–	4
2	M	51	Bethesda III	miFTC	30–50	Capsular	22	No	pT2Nx	+	+	+	–	–	1.9

SCR-FTC; follicular thyroid carcinoma with signet ring cell morphology

*M* male, *FNA* fine needle aspiration biopsy, *miFTC* minimally invasive follicular thyroid carcinoma

*ETE* extrathyroidal extension

The diagnosis was consistent with a minimally invasive follicular thyroid carcinoma with signet ring cell morphology according to the WHO 2017 classification of endocrine tumors [1]. The tumor was excised with negative margins, and no extrathyroidal extension was seen. The pTNM stage was pT2Nx (Table 1). Cytological re-investigation of the preoperative FNA biopsy material did pinpoint scarce subsets of tumor cells exhibiting a tendency of nuclear marginalization, but noticeably no clear-cut signet ring cell phenotypes.

Using next-generation sequencing (NGS) analysis, no somatic hotspot mutations were found among the 22 genes analyzed, and the tumor was wildtype for both positions C228 and C250 of the *TERT* promoter. However, two fairly common intronic single nucleotide polymorphisms (SNPs) were detected, one in *DDR2* and one in *SMAD4*, both predicted to be non-deleterious using a panel of in silico prediction software (Table 2). Moreover, using real-time PCR, no *ETV6-NTRK3* fusion transcripts were detected (data not shown).

**Table 2:** Genetic findings using the Oncomine panel for both SRC-FTCs

							In silico prediction						
Case no.	Gene	Variant	Chromato-gram allele frequency	Location / transcript	dbSNP	Global MAF	Predict SNP2	CADD	DANN	FATHMM	FunSeq2	GWAVA	Tumoral expression (IHC)*
1	*DDR2*	c.1505-20C > T	50%	Intronic: NM_006182.2	rs3738807	0.1048	N	N	N	N	N	D	n.d.
1	*SMAD4*	c.955 + 58C > T	50%	Intronic: NM_005359.5	rs948588	0.06	N	N	N	N	D	D	Positive
2	*ALK*	c.3451-30C > T	47%	Intronic: NM_004304.4	rs745932805	0.00002	N	N	D	N	D	N	Focal/weak
2	*PTEN*	c.209 + 11 T > G	69%	Intronic: NM_000314.6	Not reported	0.0	D	D	D	D	D	D	Positive

dbSNP; The Short Genetic Variations database

*MAF* minor allele frequency as listed by the gnomAD (Genome Aggregation Database)

*IHC* immunohistochemistry

*N* neutral, *D* deleterious

*n.d*. not determined

The patient was discussed at a tumor board conference and was planned for a right-sided completion lobectomy and subsequently radioiodine ablation (RAI) with a total dose of 1,1 G-Becquerel (GBq). The right thyroid lobe displayed a physiological C cell hyperplasia, but no tumors.

### Case 2

**Fig. 2:**
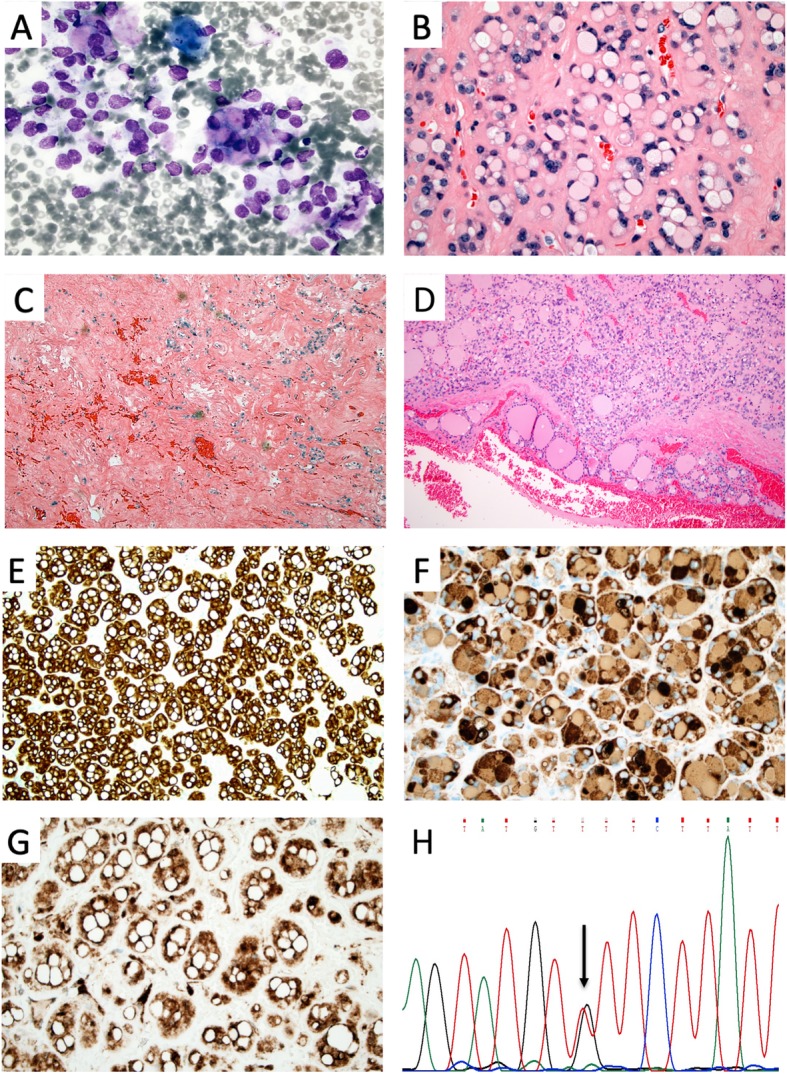
Cytological and histological illustrations of case 2. **a** Cytological preparation from the thyroid lesion at × 600 magnification depicting thyrocytes with large, irregular nuclei with suggestive nuclear marginalization in subsets of cells. The vast majority of cells were without this phenomenon, and no evident signet-ring cell phenotype was observed. **b** H&E staining of the thyroid tumor at × 400 magnification, showing thyrocytes with signet ring cell morphology in subsets (30–50%) of tumor cells. **c** Central parts of the lesion displayed a dense fibrous meshwork with focal hemorrhages and only sparsely found tumor cells. **d** One of several areas with capsular invasion, × 100 magnification. **e-g** Immunohistochemical analyses at × 400 magnification; Cytokeratin MNF116 cytoplasmic stain (**e**), thyroglobulin cytoplasmic stain, including subsets of vacuoles (**f**) and PTEN nuclear and cytoplasmic staining (**g**). **h:** Chromatogram displaying the c.209 + 11 T > G *PTEN* mutation in leukocyte DNA. The red line represents the wildtype thymine (T) and the black line represent the mutant guanine (**g**) at position c. 209 + 11

The second patient is a 51-year old male of Middle-Eastern origin, presenting with diabetes and hypertension. He has no family history of thyroid related disease. In conjunction to CT scan investigations that led to the discovery of an aneurysm of the ascending aorta, the radiologist reported a 20 mm large nodule in the caudal aspect of the right thyroid lobe. An initial FNA biopsy was inconclusive, but re-biopsy was consistent with a follicular lesion of undetermined significance (Bethesda III), with follicular-patterned, cytoplasmic-rich cells with focal intra-cytoplasmic presence of pink, dot-like amorphous deposits. In addition, subsets of cells displayed a tendency of marginalization of the nuclei (Fig. 2a). The patient underwent a right-sided hemithyroidectomy. The excided thyroid lobe displayed a weight of 12 g and measured 42 × 31 × 19 mm. At gross examination, an encapsulated tumor measuring 22 × 19 × 12 mm with a tan to yellowish cut surface was observed. Macroscopically, there were areas in which the tumor engaged the capsule.

The histological examination revealed a circumscribed lesion consisting of tumor cells with a principal micro-follicular growth pattern. The tumor cells were seen with small, relatively monomorphic nuclei with a homogenous chromatin and a punctate nucleolus, and a large subset of tumor cells exhibited a signet ring cell phenotype with a prominent vacuolization of the cytoplasmic compartment with a peripheral marginalization of the tumor nuclei (Fig. 2b). Areas with central degeneration consisting of a fibrous and hypocellular stroma were also observed (Fig. 2c). No nuclear features of papillary thyroid carcinoma were seen. No mitoses or areas with tumor necrosis were observed. Several areas with capsular invasion were noted (Fig. 2d). The tumor was devoid of extrathyroidal extension, and surgical margins were negative.

Immunohistochemical analyses confirmed the tumor as thyroid-derived, as the cells were uniformly positive for cytokeratin MNF116, PAX8 and TTF1 (Fig. 2e, Table 1). The majority of the vacuoles stained positive for thyroglobulin, indicating that the distension of the cytoplasmic compartment at least to some extent stems from inappropriate thyroglobulin accumulation (Fig. 2f). PTEN immunohistochemistry was also positive (Fig. 2g). Negative immunoreactivity was seen for chromogranin A, calcitonin, MUC2, MUC4, MUC5AC, MUC6 and CDX2. The Ki-67 index was 1.9%. The cells were also negative when assessed with a periodic-acid-schiff (PAS) stain.

The final diagnosis was a minimally invasive follicular thyroid carcinoma with signet ring cell morphology, pT2Nx. Other areas of the thyroid lobe exhibited features of Hashimoto’s thyroiditis.

Two variants were detected in somatic DNA: an unusual intronic SNP in *ALK* (rs745932805) as well as a previously unreported intronic variant in *PTEN* (Table 2, Fig. 2h). The *PTEN* variant (chr10: 87925568 T > G, c.209 + 11 T > G) is located in intron 3 of the main *PTEN* transcript, a region in which similar alterations has been judged as pathogenic and coupled to the skipping of exons 3 and/or 4 in subsets of Cowden syndrome patients [12]. Sanger sequencing analyses of histologically normal thyroid DNA from FFPE material as well as leukocyte DNA from blood preoperatively drawn from the patient confirmed the variant as constitutional. Since the variant is not listed in the SNP repositories (dbSNP, 1000 Genomes, and the Exome Variant Server), we consider this a *PTEN* intronic mutation. Further analysis with a panel of in silico prediction softwares verified the mutation as deleterious (Table 2). Using Mutation Taster, the mutation was not presumed to abrogate any potential splice sites, but the nucleotide change was predicted to create new transcription factor binding sites for 33 transcription factors (TFs) and disrupt the assumed binding site for 13 additional TFs (Additional file 1: Table S1). Several TFs with an altered binding site display a known coupling to thyroid tumorigenesis, including CREB1, FOXA1, YY1 and RXRA to name a few [13–16]. As the auxiliary immunohistochemical analysis of the patient’s FTC displayed uniform PTEN immunoreactivity in the tumor cells, this indicates that the overall expressional level of PTEN is retained.

The tumor was wildtype for both positions C228 and C250 of the *TERT* promoter, as mutations at these sites have been noted as a reliable prognostic indicator of future recurrences in follicular thyroid carcinoma [17, 18]. Moreover, using real-time PCR, no *ETV6-NTRK3* fusion transcripts were observed (data not shown).

In the ensuing tumor board conference, the patient was planned for a left-sided completion lobectomy and radioiodine ablation (RAI) with 1,1 G-Becquerel (GBq). The left thyroid lobe displayed features of Hashimoto’s thyroiditis and a cholesterol granuloma, but no tumors.

For both cases, the immunohistochemical stainings were performed in an accredited pathology laboratory using a Ventana Benchmark Ultra system (Ventana Medical Systems, Tucson, AZ, USA). All stainings were assessed by conventional light microscopy by experienced endocrine pathologists (AH and CCJ).

The Oncomine Solid Tumor Panel (Ion Torrent S5, Hi-Q Chef; Thermo Scientific) was used to screen DNA extracted from formalin-fixated paraffin-embedded (FFPE) tissues for > 1800 cancer related mutations within the following genes: *EGFR, KRAS, NRAS, PIK3CA, BRAF. ALK, ERBB2, ERBB4, FGFR1, FGFR2, FGFR3, MET, DDR2, AKT1, PTEN, MAP 2 K1, STK11, NOTCH1, CTNNB1, SMAD4, FBXW7* and *TP53*. The input DNA required was 10 ng.

For *TERT*, bi-directional Sanger sequencing of the promoter region interrogating the two mutational hotspots C228T and C250T was performed using the Genetic Analyzer 3500, Applied Biosystems, CA, USA [17] and a conventional protocol in a clinically accredited setting.

For the *ETV6-NTRK3* fusion analyses, RNA was extracted from the FFPE material using the Maxwell®16 MX3031 instrument with Maxwell®16 FFPE LEV RNA Purification Kit from Promega (Fitchburg, WI, USA). The quality and quantity of the RNA was estimated through Nanodrop technology (Nanodrop technologies, Wilmington, DE, USA). cDNA was subsequently synthesized using the High-Capacity cDNA reverse transcription Kit (Applied Biosystems, Foster City, CA, USA). Real-time PCR (TaqMan) was performed in duplicate (ABI, SDS 7000 Applied Biosystems, Foster City, CA, USA) targeting the *ETV6-NTRK3* fusion transcript corresponding to the chromosomal translocation t (12;15)(p13;q25).

Single nucleotide variants/polymorphisms for all cases were evaluated through the Predict SNP^2^ platform, in which the variant is classified as either “neutral”, “deleterious” or “unknown” using six different integrated prediction tools: Predict SNP^2^, Combined Annotation Dependent Depletion (CADD), Deleterious Annotation of Genetic Variants using Neural Networks (DANN), Functional Analysis through Hidden Markov Models (FATHMM), FunSeq2 and Genome-Wide Annotation of Variants (GWAVA) [19]. Extended analyzes for the *PTEN* variant were performed using the Mutation Taster function [20].

## Discussion and conclusions

Rare morphological variants of thyroid cancer are important to characterize from both a clinical and molecular perspective, so that an eventual coupling to underlying genetics and patient prognosis can be made. This is exemplified by the association between specific histological subtypes in papillary thyroid carcinoma and prognostic features [1]. Although the current WHO classification of endocrine tumors recognizes the SRC subtype of FTC, no information regarding the underlying genetic events and prognosis is presented – making it hard for clinicians to recognize if the SCR-FTC diagnosis should imply specific considerations in the clinical management of the individual patient.

SRC-FTC is an extremely rare entity – and drawing conclusions based on the scarce amount of cases presented in the scientific literature is challenging. Even so, a few similarities between our two cases and previously published thyroid tumors with SRC features were observed. Firstly, it seems as not all preoperative FNA biopsies are able to pinpoint the SRC morphology, including our two cases [5, 6]. Instead, the pinpointing of the SRC features was only evident after histopathological examination. This is possibly due to the fact that the SRC morphology was only observed in approximately half of the tumor cell population (Table 1), which mirrors previous findings in which SRC cells only lined parts of the follicles [6]. Indeed, only after reinvestigation of the cytology specimen, a tendency of marginalization of the nuclei was seen for a subset of tumor cells. Moreover, the cell-sparse, degenerative stromal features seen in both cases might also complicate the analysis. On histological investigation, the distended cytoplasm was positive for thyroglobulin for both cases, indicating aberrant accumulation of this protein as part of the underlying cause of the signet ring phenotype. Secondly, both our cases displayed stromal degeneration, with central areas of the tumor presenting with a dense fibrosis, sparsely populated by stromal cells and few tumor cells. Findings of a desmoplastic tumoral stroma have been previously reported in SRC tumors of the thyroid, indicating this phenomenon as a general part of the tumoral phenotype [6].

We found a previously unreported, germline mutation in the intron 3 of the tumor suppressor gene *PTEN* in case 2. Mutations in this intronic region has previously been coupled to Cowden syndrome, as a result of aberrant splicing and exon skipping [12]. Our variant is not thought to disrupt a splice site, but still predicted to be deleterious by in silico analyses. The retained tumoral PTEN immunoreactivity partly argues against an inactivating genetic event, but since the mutation was intronic, the true pathogenicity of this mutation could remain unnoticed by immunohistochemistry. The patient has no previous family history indicative of Cowden syndrome-related tumors, suggesting that the variant might not cause this disease phenotype, alternatively occurring de novo or with a reduced penetrance. DNA from additional family members was not available. Interestingly, an FTA with SRC features harboring a deleterious *PTEN* missense mutation has previously been reported in a case series of four SRC-FTAs [6]. Therefore, it seems likely that *PTEN* mutations could constitute a molecular aberrancy underlying the development of subsets of SRC thyroid tumors.

Regarding prognostication of SRC-FTC, little is known. A previous case of SRC-FTC with capsular and angio-invasive properties displayed a 12-month disease-free survival [5]. In our study, both SCR-FTCs displayed capsular invasion, but no areas with lymphovascular invasion were observed – representing a minimally invasive subtype. Moreover, the Ki-67 proliferation indexes were fairly low and no *TERT* promoter mutations were detected. In all, these factors might indicate a low risk of future recurrences – but careful monitoring is nevertheless justified given the lack of scientific literature covering this very infrequent histological subtype.

We conclude that SRC-FTCs are exceedingly rare, with subsets of cases displaying mutations in genes involved in thyroid tumorigenesis. The tumors may elude correct characterization on preoperative cytological examination based on the tumoral blend of SCR cells and conventional tumor cells. Although based on few cases, the prognosis is believed to be favorable, or at least on par with conventional FTCs.

## Supplementary information

**Additional file 1: Table S1.** In silico prediction of transcription factor (TF) binding site alterations based on the *PTEN* intronic mutation c.209 + 11 T > G in case 2 (XLSX 25 kb)

## Data Availability

All data generated or analyzed during this study are included in this published article and its supplementary information files.

## Abbreviations

CADD: Combined Annotation Dependent Depletion
CT: Computed tomography
DANN: Deleterious Annotation of Genetic Variants using Neural Networks
FATHMM: Functional Analysis through Hidden Markov Models
FFPE: Formalin-fixated paraffin-embedded
FNA: Fine needle aspiration
FTA: Follicular thyroid adenoma
FTC: Follicular thyroid carcinoma
GBq: Giga-Becquerel
GWAVA: Genome-Wide Annotation of Variants
NGS: Next-generation sequencing
NIFTP: Noninvasive follicular thyroid neoplasm with papillary-like nuclear features
PAS: Periodic-acid-schiff
PTC: Papillary thyroid cancer
RAI: Radioiodine ablation
SNP: Single nucleotide polymorphisms
SRC-FTC: FTC with signet ring cell morphology
TF: Transcription factor
